# Relationship between Tobacco, *cagA* and *vacA i1* Virulence Factors and Bacterial Load in Patients Infected by *Helicobacter pylori*

**DOI:** 10.1371/journal.pone.0120444

**Published:** 2015-03-20

**Authors:** Miguel Santibáñez, Estefanía Aguirre, Sofía Belda, Nuria Aragones, Jesús Saez, Juan Carlos Rodríguez, Antonio Galiana, Javier Sola-Vera, Montserrat Ruiz-García, María Paz-Zulueta, Raquel Sarabia-Lavín, Alicia Brotons, Elena López-Girona, Estefanía Pérez, Carlos Sillero, Gloria Royo

**Affiliations:** 1 Departamento de Salud Pública, Universidad de Cantabria, Santander, Spain; 2 IDIVAL-Instituto de Investigación Marqués de Valdecilla, Santander, Spain; 3 Microbiology S. Elche University General Hospital, Elche (Alicante), Spain; 4 CIBER in Epidemiology and Public Health (CIBERESP), Madrid, Spain; 5 Environmental and Cancer Epidemiology Unit, National Center of Epidemiology, ISCIII, Madrid, Spain; 6 Digestive Medicine S. Elche University General Hospital, Elche (Alicante), Spain; 7 Microbiology S. Alicante University General Hospital, Alicante, Spain; 8 Miguel Hernández University, Elche, Spain; 9 Departamento de Enfermería, Universidad de Cantabria, Santander, Spain; Indian Institute of Science, INDIA

## Abstract

**Background and Aim:**

Several biological and epidemiological studies support a relationship between smoking and *Helicobacter pylori (H*. *pylori)* to increase the risk of pathology. However, there have been few studies on the potential synergistic association between specific *cagA* and *vacA* virulence factors and smoking in patients infected by Helicobacter pylori. We studied the relationship between smoking and *cagA*, *vacA i1* virulence factors and bacterial load in *H*. *pylori* infected patients.

**Methods:**

Biopsies of the gastric corpus and antrum from 155 consecutive patients in whom there was clinical suspicion of infection by *H*. *pylori* were processed. In 106 patients *H*. *pylori* infection was detected. Molecular methods were used to quantify the number of microorganisms and presence of *cagA* and *vacA i1* genes. A standardized questionnaire was used to obtain patients’ clinical data and lifestyle variables, including tobacco and alcohol consumption. Adjusted Odds Ratios (OR_adjusted_) were estimated by unconditional logistic regression.

**Results:**

*cagA* was significantly associated with active-smoking at endoscope: OR_adjusted_ 4.52. Evidence of association was found for *vacA i1* (OR_adjusted_ 3.15). Bacterial load was higher in active-smokers, although these differences did not yield statistical significance (median of 262.2 versus 79.4 copies of *H*. *pylori* per cell).

**Conclusions:**

The association between smoking and a higher risk of being infected by a virulent bacterial population and with higher bacterial load, support a complex interaction between *H*. *pylori* infection and environmental factors.

## Introduction

Interaction between *Helicobacter pylori* (*H*. *pylori*) and gastric mucosa is a complex phenomenon and as yet little understood. Though in most cases this microorganism does not cause any symptomatic pathology, in some cases it causes serious lesions in the mucosa, being the main risk factor for gastric cancer. This different patterns of evolution may be related to the host’s characteristics (genetic, immunological), environmental exposures (socio-sanitary and life-style factors), and also to certain characteristics of the microorganism [1].

Many virulence genes have been described in *H*. *pylori*, and attempts have been made to associate them with different diseases. One of the most widely studied factors is the virulence *cagA* gene. This gene encodes the CagA protein, which can be directly injected by the bacterium into colonized epithelium via a type IV secretion system, leading to cellular morphological, anti-apoptotic and proinflammatory effects responsible in the long-term (years or decades) for ulcer and cancer [2].

Other virulence gene described in *H*. *pylori* is *vacA*, which encodes VacA, a vacuolating cytotoxin initially identified for its ability to induce vacuolation in epithelial cells [1,3–5]. Though all strains contain *vacA*, this gene contains three distinct segments that exhibit variation starting within the amino terminus. These areas of variation are broadly defined as the signal (*s*), intermediate (*i*), and middle (*m*) regions, and two or more primary variants have been described for each region: *s1* and *s2* for the signal region, *i1*, *i2*, and *i3* for the intermediate region, and m1 and *m2* for the middle region [1,5–8]. Several studies have linked strains carrying the *s1-m1* allele of the toxin to more-severe disease outcomes, since these strains show the strongest vacuolating activity to the broadest range of cells [9]. The *i* region is positioned between the *s* and *m* regions and is the most recent region to be described. The *i1* variants of *vacA* have been shown to have stronger vacuolating activity than toxins containing the *i2* regions and recently, the *i* region has been suggested to be a better predictor of disease severity than either the *s* or *m* region, though the *i* region appears to covary with the *s* and *m* regions. This means that the more toxic *i1* region is often associated with *s1-m1* [7].

In addition, some studies have also described an association between the presence of a greater number of microorganisms and more serious gastric lesions with independence of these virulence factors [10,11].

Regarding the host’s characteristics, several biological [12,13] and epidemiological studies support a relationship between smoking and *H*. *pylori* to increase the risk of pathology [14–17].

However, there have been few studies on the potential synergistic association between specific *cagA* and *vacA* virulence factors and tobacco use (chewing or smoking) [18,19,20] and to the best of our knowledge, there are not published studies on the relationship between tobacco use and *H*. *pylori* bacterial load.

Our aim was to study the relationship between smoking and *cagA*, *vacA i1* virulence factors and bacterial load in *H*. *pylori* infected patients.

## Material and Methods

### Ethics Statement

All subjects were informed of the study objectives and gave their informed consent prior to their inclusion in the study. The research protocol was approved by the local ethics and research committees of the participating hospital: "Comité de bioética asistencial (CBA) Dpto Salud 20-H. General" and "Comité ético de investigación clínica (CEIC) del Hospital General Universitario de Elche". Patient records/information was anonymized and de-identified prior to analysis."

### Design and participants

Details of this study have been published elsewhere [11,21,22]. Biopsies of the gastric corpus and antrum from 155 consecutive patients in whom there was clinical suspicion of infection by *H*. *pylori* were processed: duodenal ulcer (n = 67), duodenitis (n = 19), gastric ulcer (n = 36), gastritis (n = 5), dyspepsia (n = 11), family history of gastric cancer (n = 3), and eradication failure (n = 14).

Prospective study including biopsies of the gastric corpus and antrum obtained from the first 155 symptomatic patients who consecutively attended the Endoscopy Unit of Elche University General Hospital for an upper digestive tract endoscopy between January 2009 and June 2010, in whom investigation of *H*. *pylori* was indicated

### Data sources and variables

#### Measures

A standardized questionnaire was used to obtain patients’ clinical data and lifestyle variables, including tobacco and alcohol consumption. Smoking habit was classified at the time of endoscope as ‘active smokers’ versus ‘non active smokers’. An ‘active smoker’ was defined as an smoker at the time the endoscope was performed or up to a week previously, irrespective of the number of cigarettes smoked per day and for how many years. In a second step, a never smoker was defined as someone having smoked fewer than 100 cigarettes ever or less than one cigarette per day for one year. A former smoker was defined as someone having stopped smoking 1 or more years before the endoscope.

S1 Table shows the age, gender, clinical characteristics and lifestyles of the 155 patients in relation to smoking status at endoscope.

*H*. *pylori* infection was detected in 106 patients according to a validated real-time polymerase chain reaction (PCR) [21] that also allows quantifying the number of microorganisms per cell. In 96 and 73 out of these 106 patients, the remains of biopsies allowed us to determine *cagA*, and *vacA i1* genes respectively.

#### *cagA* determination and *vacA i1* region sequencing

Detection of the *cagA* gene was done using a real-time PCR system designed in our laboratory [11,21]. After that, we also designed a nested PCR for the amplification of the intermediate region (i) the gene *vacA*, permitting the classification of the gene fragment at i1, i2 and i3 through three clusters (A, B and C) [5]. Primers used in the study of the virulence factors of *H*. *Pylori* are shown in S2 Table.

Quantification of the number of microorganisms. The quantification of the microorganisms was carried out by amplifying a fragment of the urease gene; the results were expressed as the quotient of the number of microorganisms and the number of human cells in each gastric sample [21].

### Statistical analyses

A non-parametric test (U Mann Whitney for independent samples) was used to compare the number of microorganisms in the biopsy and smoking status. In order to estimate the association between *cagA* and *vacA i1* virulence factors, and bacterial load and tobacco smoking, adjusted odds ratios (OR_adjusted_) and 95% confidence intervals (95%CI) were calculated using unconditional logistic regression. All regression models included as covariates sex, age (< = 50 years, >50 years), average of pure ethanol (g/day) (never, moderate, high), consumption of PPI in the days prior to endoscopy (yes/no), consumption of NSAID (yes/no), and clinical presentation of UDTH (yes/no) entered as indicator variables.

Tests for trend in the ORs across exposure strata were calculated for tobacco smoking ordinal categorised (never, former, current smoker) by using logistic models that included categorical terms as continuous variables in a model with all the potential confounders. For trend-tests, we used the likelihood ratio test statistic with one degree of freedom.

The level of statistical significance was set at 0.05 and all tests were two-tailed. All analyses were performed with SPSS v.21.0.

## Results

Table 1 presents the association between *cagA* and tobacco consumption. There was a statistically significant association between *cagA* positive status and active smoking at endoscope (OR_adjusted_ 4.52; 95%CI 1.28–15.98). Table 2 shows the associations between *vacA i1* region status and active smoking at endoscope. *vacA i1* positivity is associated with smoking, though this association does not attain statistical significance (crude OR 2.69; 95% CI 0.92–7.92).

**Table 1:** Association between *cagA* status and tobacco smoking.

	N *cagA* neg	N *cagA* pos	OR	95%	CI	OR_adjusted_ ^a^	95%	CI
**Smoking Status at endoscope**								
Non active smokers	34	31	1.00			1.00		
Active smokers	8	23	3.15	1.23	8.07	4.52	1.28	15.98
**Smoking Status**								
Never	22	23	1.00			1.00		
Former smoker	12	8	0.64	0.22	1.86	0.36	0.09	1.53
Current smoker	8	23	2.75	1.02	7.43	3.24	0.84	12.47
*p-value for linear trend*				*0*.*067*			*0*.*123*	

^a^Adjusted for sex, age (< = 50 years, >50 years), average of pure ethanol (g/day) (Never, moderate, high), consumption of proton pump inhibitors in the days prior to endoscopy (Yes/No), consumption of non-steroid anti-inflammatory drugs (Yes/No), and clinical presentation of upper digestive tract haemorrhage (Yes/No).

**Table 2:** Association between *vacA i1* intermediate region status and tobacco smoking.

	N *vacA Region_i1*neg	N *vacA Region_i1* po*s*	OR	95%	CI	OR_adjusted_ ^a^	95%	CI
**Smoking status at endoscope**								
Non active smokers	21	26	1.00			1.00		
Active smokers	6	20	2.69	0.92	7.92	3.15	0.74	13.39
**Smoking status**								
Never	12	21	1.00			1.00		
Former smoker	9	5	0.32	0.09	1.17	0.38	0.07	2.13
Current smoker	6	20	1.91	0.60	6.05	2.20	0.46	10.58
*p-value for linear trend*				*0*.*356*			*0*.*351*	

^a^Adjusted for sex, age (< = 50 years, >50 years), average of pure ethanol (g/day) (Never, moderate, high), consumption of proton pump inhibitors in the days prior to endoscopy (Yes/No), consumption of non-steroid anti-inflammatory drugs (Yes/No), and clinical presentation of upper digestive tract haemorrhage (Yes/No).

Finally, bacterial load was very asymmetric and presented a high variability. The number of copies of *H*. *pylori* per cell in antrum according to real-time PCR was higher in active-smokers: Median = 262.2 [Interquartile Range = 2079.0] than in no active-smokers: Median = 79.4 [Interquartile Range = 890.8], although these differences were not statistically significant *(p = 0*.*35)*. Positive associations without yielding statistical significance were found between active smoking and the number of microorganisms when the bacterial load was divided into above and below median: OR_adjusted_ 2.26 (95% CI 0.76–6.75) (see Table 3).

**Table 3:** Association between bacterial load and tobacco smoking.

	*H. pylori* bacterial load							
	*≤ Median (N)*	*> Median (N)*	OR	95%	CI	OR_adjusted_ ^a^	95%	CI
**Smoking Status at endoscope**								
Non active smokers	39	33	1.00			1.00		
Active smokers*	14	20	1.69	0.74	3.85	2.26	0.76	6.75
**Smoking status**								
Never	28	21	1.00			1.00		
Formersmoker	11	12	1.45	0.54	3.93	1.29	0.34	4.90
Current smoker	14	20	1.90	0.78	4.62	2.47	0.76	8.06
*p-value for linear trend*				*0*.*150*			*0*.*137*	

^a^Adjusted for sex, age (< = 50 years, >50 years), average of pure ethanol (g/day) (Never, moderate, high), consumption of proton pump inhibitors in the days prior to endoscopy (Yes/No), consumption of non-steroid anti-inflammatory drugs (Yes/No), and clinical presentation of upper digestive tract haemorrhage (Yes/No).

For both *cagA* and *vacA i1* virulence factors and bacterial load, a dose response pattern was obtained when non active smokers were divided into never and former smokers (current smokers were at higher risk than former smokers).

## Discussion

Overall, in our study, when restricting to *H*. *pylori* infected patients, active smokers were at a higher risk of colonization by *cagA* positive virulent strains at the time of endoscope. Although results did not yield statistical significance, active smoking was also associated to *vacA i1* virulent strains and higher bacterial loads.

First contact with *H*. *pylori is* documented by a transmission of the bacteria within families, predominantly in the early childhood [23,24]. However, it has been found that the genome of *H*. *pylori* changes continuously during chronic colonization of an individual host with mixed strains by importing small pieces of foreign DNA from other strains during persistent or transient mixed infections [25–27].

The co-colonization of *H*. *pylori* could permit inter-strain DNA transfer, which could speed up the emergence of new strains/subpopulations that are better adapted or more virulent in a given human host than the initial infecting strains [25, 28–30].

The higher bile salt reflux and gastric bile salt and the lower concentrations of vitamin C in gastric juices [12,13] in smokers in comparison with non-smokers support a biological plausibility on the relationship between smoking and a higher prevalence of new more virulent strains with higher bacterial loads.

A growing number of studies have begun to suggest that *vacA* and *cagA* interact in such a way as to affect disease severity [31–34]. In a previous work we found that the *cagA* gene was associated with high bacterial load with a clear, statistically significant dose-response relationship between load and presence of *cagA*. These data suggest that both parameters combine to produce clinically significant lesions, as compared with strains of low virulence that multiply very little in the gastric mucosa and so do not cause significant damage [11].

Our present data suggest that smoking could be a synergistic factor, interacting with both bacterial load and *H*. *pylori* virulence factors. Published studies would support an interaction between high virulence factors and smoking to increase the risk of intestinal metaplasia [14,18] and gastric cancer [15–17,19]. Recently, an association between *cagA* and other types of tobacco use such as tobacco-chewing has been also documented [20].

Since a synergistic effect seems plausible, the presence of high virulence strains of *H*. *pylori* should be included as a confusion variable and as a term of interaction in the associations traditionally established between smoking and gastric cancer [35,36].

If this hypothesised synergistic effect is firmly established, smoking cessation would be associated with a lower risk of high virulence strains infection, so the combined effect of smoking and *H*. *Pylori* trends on past and future gastric cancer incidence, could be higher than estimated by microsimulation models [37].

Up to our knowledge, this is the first study on the specific relationship between smoking and bacterial load. Since our results did not yield statistical significance, because of the asymmetry and high variability of bacterial load, further prospective studies with higher sample size and statistical power are necessaries to corroborate these findings.

Number of CagA Glu-Pro-Ile-Tyr-Ala (EPIYA)-C segments (with multiple repeats increasing the virulence) may explain, to some extent, geographic differences in the incidence of gastric cancer in Western countries. It would be interesting to go in depth in relation to the association between smoking and these specific patterns of *cagA* gene repeat sequences. Further studies are also required to continue studying the relationship between tobacco and *vacA* alleles, as well as other more recently discovered virulence factors [38,39].

In conclusion, the associations between smoking and a higher prevalence of virulent bacterial population with higher bacterial loads, support a complex interaction between the microorganism, gastric mucosa and environmental factors such as tobacco smoking, in which synergistic effects could increase gastric pathology.

## Supporting Information

S1 Table Baseline characteristics of patients as a function of Smoking Status at endoscope. ^a^SD denotes Standard Deviation ^b^Alcoholism (high alcohol consumption) was defined as a consumption of more than 80g/day in men and 60 g/day in women ^c^A Coffee/Tea Drinker at the endoscope was defined as someone having consumed at least two cups at week for at least one year. ^d^Comorbidity was defined as someone having high blood pressure, diabetes, cardiopathy, chronic obstructive pulmonary disease, or kidney failure.
(PDF)

S2 Table Primers used in the study of the virulence factors of *H*. *pylori*. (PDF)
