# Correction: Relationship between Tobacco, *IcagA* and *vacA i1* Virulence Factors and Bacterial Load in Patients Infected by *Helicobacter pylori*

**DOI:** 10.1371/journal.pone.0126540

**Published:** 2015-04-15

**Authors:** The PLOS ONE Staff

There are errors in the Funding section. The complete, correct funding information is as follows: This research was supported by grants from the Fundación Bienvenida Navarro-Luciano Trípodi (FBNLT01/2009); the Generalitat Valenciana Proyectos Precompetitivos (GV-PP2008/317) to MRG; by Fundación para el fomento de la Investigación del Hospital General Universitario de Elche (FIBELX 08/01) to JCR; and by Instituto de Salud Carlos III http://www.eng.isciii.es/ (PI11/01403) to NA. The funders had no role in study design, data collection and analysis, decision to publish, or preparation of the manuscript.

The publisher apologizes for the error.
