# Supplementary material for: Relationship between Tobacco, cagA and vacA i1 Virulence Factors and Bacterial Load in Patients Infected by Helicobacter pylori
Source: PLoS One. 2015 Mar 20;10(3):e0120444. doi: 10.1371/journal.pone.0120444 (PMC4368826; doi:10.1371/journal.pone.0120444)
Supplement: S1 Table — aSD denotes Standard Deviation bAlcoholism (high alcohol consumption) was defined as a consumption of more than 80g/day in men and 60 g/day in women cA Coffee/Tea Drinker at the endoscope was defined as someone having consumed at least two cups at week for at least one year. dComorbidity was defined as someone having high blood pressure, diabetes, cardiopathy, chronic obstructive pulmonary disease, or kidney failure. (PDF) [file pone.0120444.s001.pdf]

S1 Table. Baseline characteristics of patients as a function of Smoking Status at endoscope.

	**Total N=155**	**column %**	**No-Active smokers N=113**	**row %**	**Active Smokers N=42**	**row %**	**p value**
Age: Mean [SD][a]	55.9 [17.2]	--	58.7 [17.2]	--	47.4 [14.4]	--	<0.001
Women	69	45%	56	81%	13	19%	0.04
Men	86	55%	57	66%	29	34%	
Alcoholism (Yes)[b]	8	5%	5	63%	3	37%	0.51
Alcoholism (No)	146	94%	107	73%	39	27%	
Coffe/Te consumption (Yes)[c]	63	41%	42	67%	21	33%	0.9
Coffe/Te (No)	40	26%	27	68%	13	32%	
Comorbidity (Yes)[d]	58	37%	49	85%	9	15%	0.02
Comorbidity (No)	78	50%	52	67%	26	33%	
PPI consumption in previous days	46	30%	39	85%	7	15%	0.06
PPI (No)	89	57%	62	70%	27	30%	
NSAID consumption in previous days	39	25%	24	62%	15	38%	0.02
NSAID (No)	96	62%	77	80%	19	20%	
Antibiotic consumption in previous days	5	3%	5	100%	0	0%	0.18
Antibiotic (No)	128	83%	94	73%	34	27%	
Upper digestive tract hemorrhage (UDTH)-Yes	78	50%	59	76%	19	24%	0.44
UDTH (No)	77	50%	54	70%	23	30%	
H. Pylori Infection (Gold standard)	85	55%	56	66%	29	34%	0.03
No-Infection	70	45%	57	81%	13	19%	
H. Pylori Infection (Real-Time PCR >0)	106	68%	72	68%	34	32%	0.04
No-Infection	45	29%	38	84%	7	16%	

[a]SD denotes Standard Deviation

[b]Alcoholism (high alcohol consumption) was defined as a consumption of more than 80g/day in men and 60 g/day in women

[c]A Coffee/Tea Drinker at the endoscope was defined as someone having consumed at least two cups at week for at least one year.

[d]Comorbidity was defined as someone having high blood pressure, diabetes, cardiopathy, chronic obstructive pulmonary disease, or kidney failure.
