# Supplementary material for: Relationship between Tobacco, cagA and vacA i1 Virulence Factors and Bacterial Load in Patients Infected by Helicobacter pylori
Source: PLoS One. 2015 Mar 20;10(3):e0120444. doi: 10.1371/journal.pone.0120444 (PMC4368826; doi:10.1371/journal.pone.0120444)
Supplement: S2 Table — (PDF) [file pone.0120444.s002.pdf]

S2 Table. Primers used in the study of the virulence factors of *H. pylori*

	cagA Gene	***vacA Gene***
F-PCR<br>Sequence-Ext_F	5-GAGCAAGCRYTAGCCGAT CTCA-3	5-ACAAGSAATACGACTTAT ACAAATCCT-3
R-PCR-1<br>Sequence-Ext_R	5-TTGTAGAATCTTTGAGCTTGTCTATCATTTG-3	5-TTTGCCACAAAT CCA GTC TGC-3
F-PCR-2<br>Sequence-Int_F	5-GTTAARAATRGTGTRAAYGG-3	5-GTTGGGATTGGGGGA ATGCCG-3
F-PCR-2<br>Sequence-Int_F	5-TTTAGCTTCTGATACCGC-3	5-TTTGAAGTGTYTATCGTG CTGTATGAAGGG-3
